# What the pediatric endocrinologist needs to know about skeletal dysplasia, a primer

**DOI:** 10.3389/fped.2023.1229666

**Published:** 2023-08-22

**Authors:** Janet M. Legare, Donald Basel

**Affiliations:** ^1^ Department of Pediatrics, University of Wisconsin School of Medicine and Public Health, Madison, WI, United States; ^2^ Department of Pediatrics, Medical College of Wisconsin, Milwaukee, WI, United States

**Keywords:** skeletal dysplasia, achondroplasia, short stature, hypochondroplasia, hypophosphatasia, osteogenesis imperfecta

## Abstract

Children with skeletal dysplasia are frequently referred to pediatric endocrinologists due to short stature. These children may present with disproportionate growth or medical histories that point to a skeletal dysplasia. This primer will discuss when to be concerned about skeletal dysplasia, the initial steps in evaluation for a skeletal dysplasia, and new therapies that are either recently approved or in development.

## Introduction

Endocrinologists are experts in growth and as such receive a large majority of the referrals for all forms of short stature. This includes patients with recognized or undiagnosed skeletal dysplasias. It is not uncommon for the term “*skeletal dysplasia*” to elicit an irrational fear of the unknown. The intent of this primer is to dispel any enigma and provide a practical approach to considering diagnosis and intervention for children suspected of carrying a diagnosis of one of the skeletal dysplasias. Inflammatory processes such as juvenile idiopathic arthritis (JIA) can also present as short stature with joint difficulties, but do not fall within the purview of this article.

The skeletal dysplasias are individually rare but collectively have a prevalence of 1:4,000 (1). There are now more than 700 skeletal dysplasias described (1). In general, symptomatic care has been the only treatment option aside from the metabolic lysosomal storage disorders, for which a subset had enzyme replacement therapy (2, 3). Recently, vosoritide was approved for increasing growth velocity in achondroplasia (4). Research in various types of collagen, COMP, FGFR3, FGF23, TGFβ, as well as other enzyme replacements are all promising to affect clinical outcomes in patients with skeletal dysplasias (5–13). Being able to recognize when a patient may have a skeletal dysplasia is key to implementing appropriate treatment. This primer will discuss when to consider a diagnosis of skeletal dysplasia, how to start an appropriate evaluation, and recent advances in treatments. Some skeletal dysplasias such as Leri Weill dyschondrosteosis and Turner syndrome do respond to growth hormone and its use is appropriate, in others, growth hormone is futile and not indicated (14, 15).

## Proportions and growth

**Figure 1:**
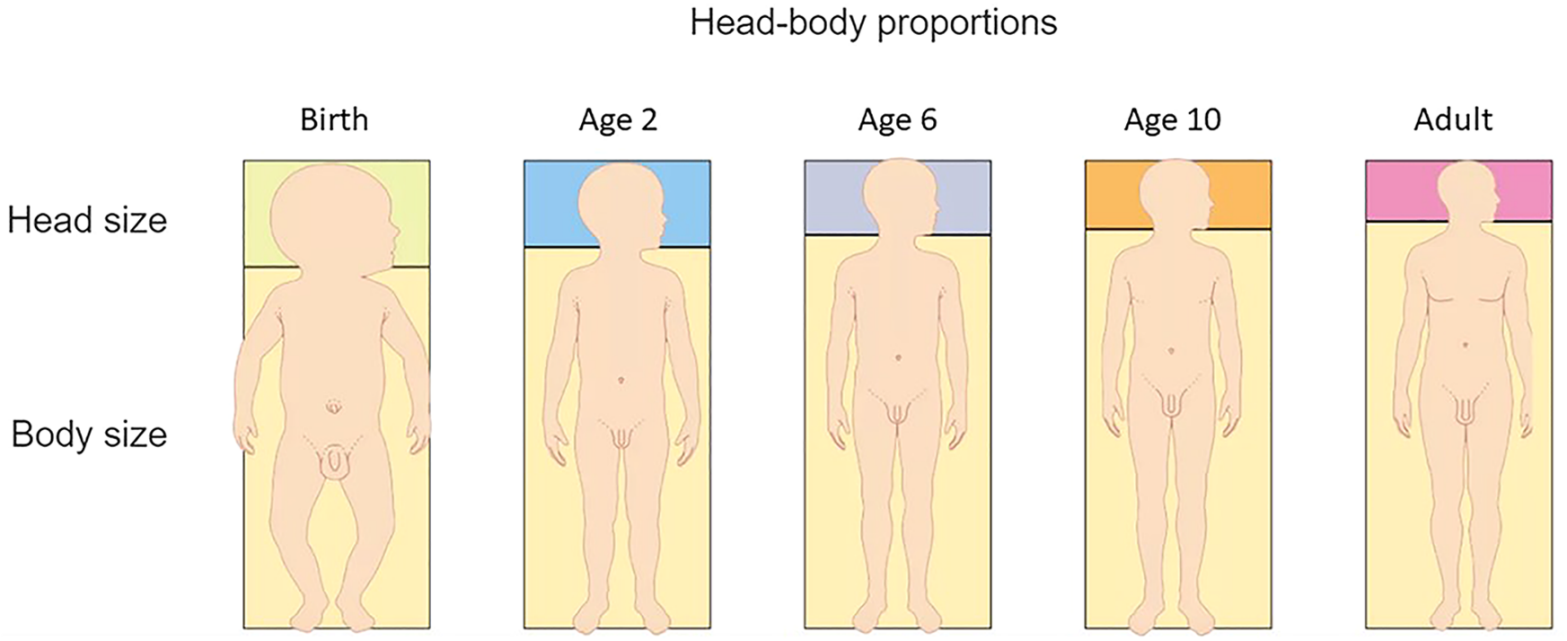
Transitioning body proportions from birth to adulthood. (Figure adapted from Dorling Kindersley www.dkfindout.com).

Children are generally disproportionate during their initial growth phase; this is most notable in babies who have larger heads and a relatively longer trunk in relation to their shorter extremities (Figure 1) (16). This becomes less noticeable at around two years of age. In infants, the midpoint of the body is 1.8 cm above the umbilicus, but at two years old it is just below the umbilicus and in adults it is at the symphysis pubis. Sitting height also changes over time. In infancy, sitting height is approximately 70% of total length whereas at 3 years old it is 57% and by puberty it is 53% (17).

Extremities are shorter at birth**,** but by 10 years of age, the wingspan should be approximately the same as the height. The head becomes a smaller proportion of the body surface area and height as a child grows. The upper to lower segment ratio (measured from the symphysis pubis) should be about 1.0 at 11 years of age and hands should come down to approximately 1/3–1/2 way down the thighs when standing. The proximal segment, (humerus/femur) is longer than the middle segment (forearm and lower leg) which is subsequently longer than the distal segment (hands and feet) (16–18). When these proportions differ, the child looks disproportionate. This can occur when limbs are shorter than typical, trunk is shorter than typical, or a combination of the two.

Bone may appear to be an inert structural component of our musculoskeletal system but it is a dynamic tissue, constantly being remodeled in response to both mechanical and physiological pressures. This function is crucial for maintaining bone strength and calcium/phosphate homeostasis. In order for bone to form normally, we need to consider both the embryological pathways for normal bone development and the homeostatic regulation of normal, healthy bone. For the purposes of this discussion, we will limit concepts to those relevant for endochondral ossification which account for the majority of the bones in the body.

Endochondral ossification begins in early fetal development, mesenchymal stem cells transform into osteoprogenitor lineages and finally into osteocytes or chondrocytes through the action of numerous transcription factors. Many of these transcription factors or receptors are causally associated with various skeletal dysplasias. It is beyond the scope of this review to include all known pathoetiological mechanisms for skeletal dysplasias, the intent is to highlight those which have targeted therapies available or in investigation.

## Skeletal dysplasias

**Figure 2:**
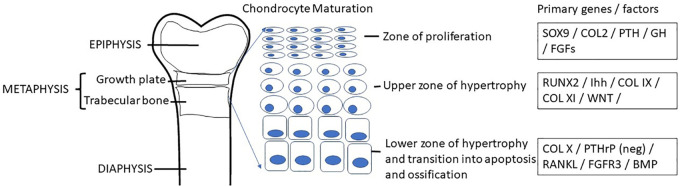
Chondrogenesis occurs within the growth plate as a result of the complex orchestration of chondrocyte proliferation, hypertrophy, secretion of matrix protein elements and apoptosis leaving osteoid which is then vascularized and ultimately mineralized by osteoblasts while osteocytes aid in the remodeling to form the functional bone structure. A coordinated network of hormones including growth hormone, glucocorticoid, thyroid hormone, estrogen, androgen, vitamin D, and leptin interact with numerous transcription factors for normal regulation of longitudinal bone growth.

Skeletal dysplasias can result in growth differences of the various elements of bone in addition to overall structure of the bone. The consistent element in skeletal dysplasias is ‘disproportion’, and this generally separates short stature from any other cause (Table 1). The epiphysis is the end of the bone on the distal end of the growth plate or physis and the metaphysis is on the proximal side of the growth plate (Figure 2). The radiological description of skeletal dysplasias typically relates to the most affected elements of the bone. Epiphyseal dysplasias predominantly affect the epiphyses, whereas metaphyseal dysplasias primarily affect the metaphyses. Various combinations such as epi-metaphyseal dysplasias if these elements are equally involved and spondylo- epi-and or metaphyseal if the vertebral bodies are affected. Involvement of the diaphysis is rare. In general, epiphyseal dysplasias result in more joint pain and a waddling gait whereas metaphyseal dysplasias result in less differences in pain in childhood. Epiphyses appear late in epiphyseal dysplasias. As endocrinologists know, bone ages are used as a standard for growth. However, bone ages are based on epiphyseal ossification of the radius, ulna, carpels, metacarpals, and phalanges. Epiphyseal dysplasias always present with a delayed bone age because epiphyseal ossification is late and abnormal.

**Table 1:** Physical features that warrant further work up for skeletal dysplasia.

• Disproportion 1. Elevated or decreased sitting height:total height 2. More than 2 cm difference between wing span and height after age 10 • Micromelia, rhizomelia, mesomelia, or acromelia • Extremity angulation—either varus or valgus • Severe scoliosis • Madelung deformity • Small ears with any cysts • Dental abnormalities—abnormal enamel, conical teeth • Facial dysmorphisms—midface hypoplasia, low set ears, large or small chin, blue sclerae, cleft palate

**Figure 3:**
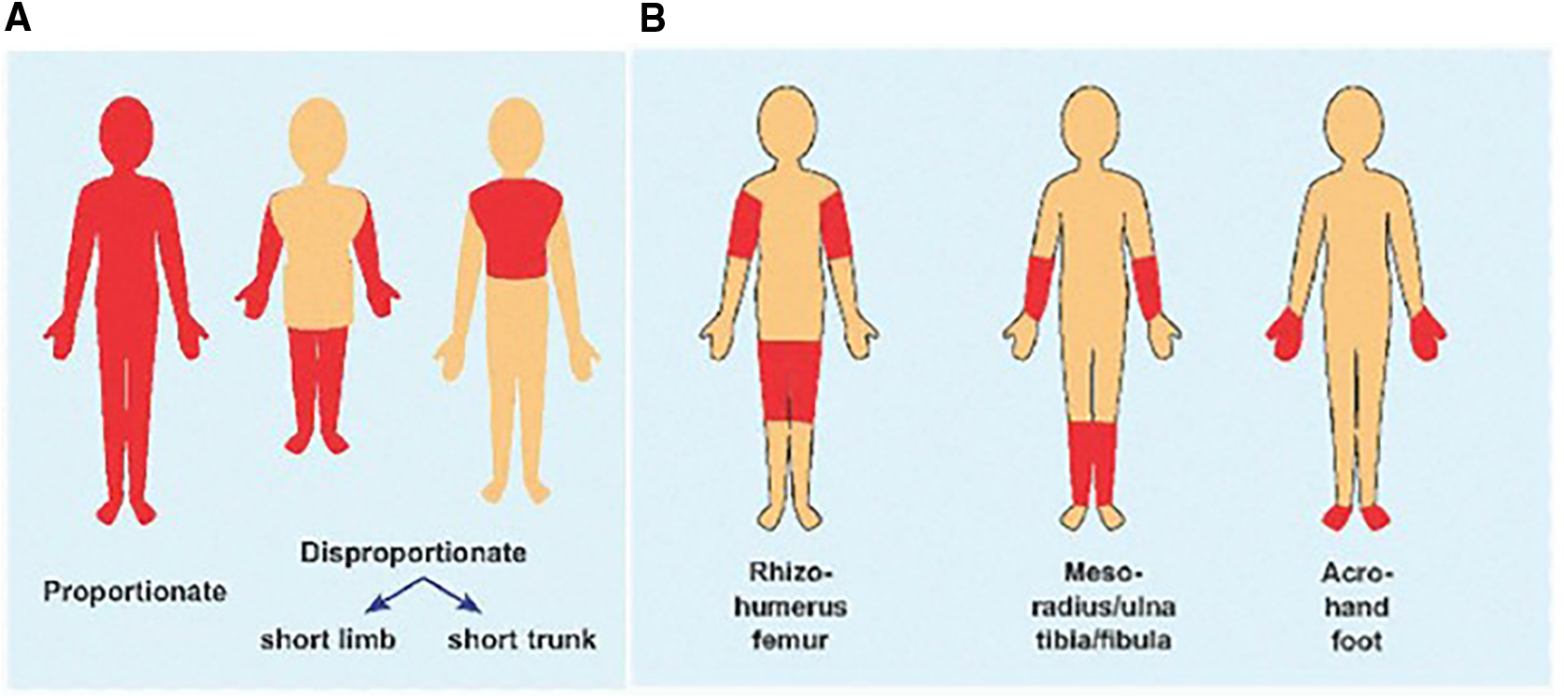
(**A**) Cartoon showing body disproportion, short limbs vs. short trunk. (**B**) Segment of limb involved for rhizomelia, mesomelia and acromelia. (Figure adapted from Musculoskeletal Key).

The following terminology is used to describe disproportion. Micromelia refers to overall shortening of the limb. Rhizomelia means shortening of the proximal segment. Mesomelia refers to shortening of the middle segment, and Acromelia refers to shortening of the distal segment. Any combination can be present and used as a clinical descriptor, e.g., acromesomelic dysplasia. Short trunked dysplasias mean a short chest and abdomen compared to the extremities, whereas long trunked dysplasias refer to a relatively long chest and abdomen compared to shortening of the extremities (Figure 3). Bone dysplasias can result in joint angulation and scoliosis (Table 1) and can vary in presentation with some children being short from birth and others falling off the length curve by 18–24 months of age. Proportions can also change throughout childhood. For example, type 2 collagenopathies and dysplasias from *TRPV4* pathogenic variants show more disproportion with short trunk and longer extremities as a child ages (19, 20). Disproportionate short stature with specific medical histories (Table 2) or family histories also point toward skeletal dysplasia.

**Table 2:** Medical history that warrants further evaluation for possible skeletal dysplasia.

• Significant myopia (high mypopia) with prescriptions worse than −5. • Retinal detachment • Hearing loss—either conductive or sensorineural • Dental caries requiring multiple caps, extractions. Late teeth eruption or loss • Family history of joint replacements under age 50 • Progressive scolsiosis • Notable tracheomalacia • Myelopathy • Early fractures • Abnormal bone density on xrays • Charcot Marie Tooth

## Common skeletal dysplasias

**Figure 4:**
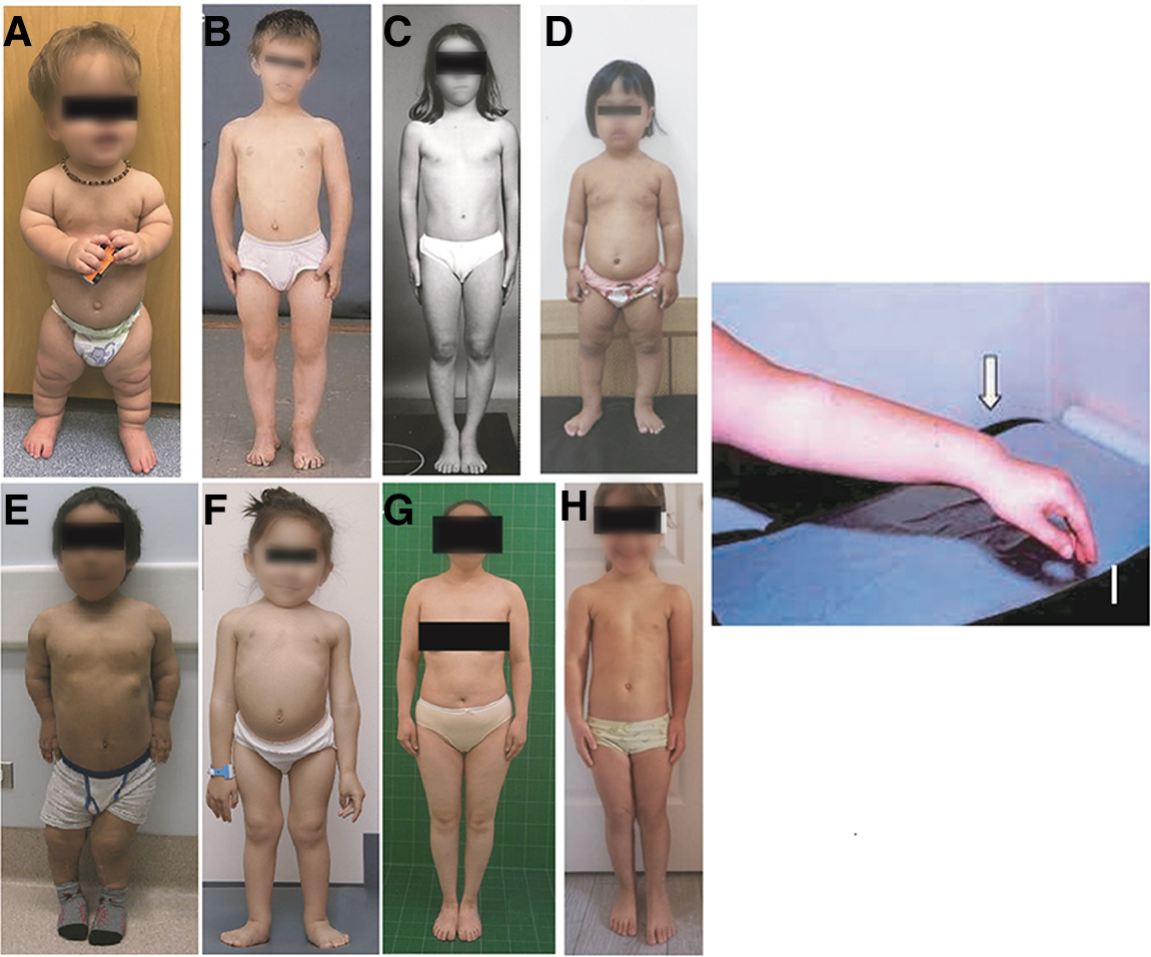
Various diagnoses of skeletal dysplasia: (**A**) achondroplasia; (**B–D**) hypochondroplasia; (**E**) Pseudoachondroplasia; (**F**) SEDC; (**G**) MED; (**H**) leri-weil dyschondrosteosis; (**I**) madelung deformity.

Long trunked/short extremity skeletal dysplasias include achondroplasia, which is the most common disproportionate short statured skeletal dysplasia, hypochondroplasia, pseudoachondroplasia, Leri-Weill dyschondrosteosis (LWD), Turner syndrome and diastrophic dysplasia. A common finding in patients with Turner syndrome or LWD is Madelung deformity at the wrist—bowing of the radius with dorsal subuxation of the distal radioulnar joint (Figure 4). Multiple Epiphyseal Dysplasia (MED) may present with very subtle disproportion with shortened extremities, but these patients frequently have a family history of early joint replacement and may also present with myopathy (21). In all of these dysplasias, the wingspan is shorter than the height or length. Historically, hypochondroplasia and MED give the pediatric endocrinologist the most difficulty given that patients may not have overt defining features. Examples of short, trunked/long extremity skeletal dysplasias include spondyloepiphyseal dysplasia congenita (SEDC), other type 2 collagenopathies, and spondylometaphyseal dysplasia Kozlowski or metatropic dysplasia. Disproportion is again evident; in SEDC it is common for a child's arms to reach down to just above their knees. Figure 4 shows various patients with skeletal dysplasias.

**Figure 5:**
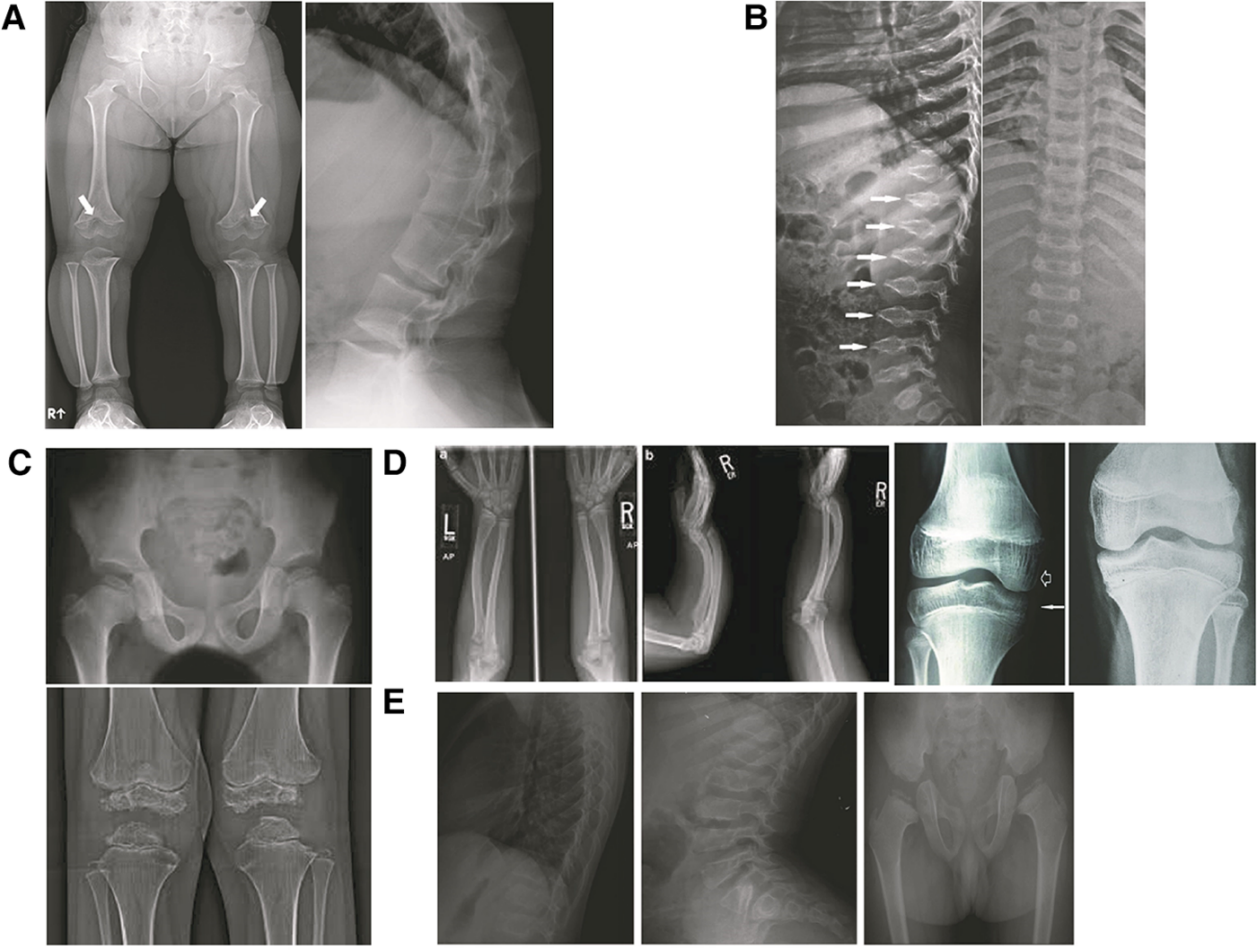
X-ray findings in various skeletal dysplasias: (**A**) achondroplasia: note chevron shape of distal femur physes, flat acetabular roof, relatively narrow sacroiliac notch, broad pelvis, metaphyseal flaring, short robust bones, short pedicles on lateral spine film; (**B**) pseudoachondroplasia: note the platyspondyly and oval vertebral bodies with anterior beaking (**C**) MED: bilateral femoral heads, distal femoral epiphyses and proximal tibial epiphyses small and ragged; (**D**) SHOX: madelung deformity, downward displacement of distal femur medial condyles; (**E**) SEDC: flattened vertebrae (platyspondyly), elongated vertebral bodies, late appearing and small femoral heads.

Chondrogenesis at the growth plate in endochondral bones results in accrual of height (Figure 2). Achondroplasia and hypochondroplasia are part of the Fibroblast Growth Factor Receptor 3 (*FGFR3*) family of diagnoses (1). FGFR3 is an important negative inhibitor of endochondral bone growth. In children with pathogenic gain of function variants in *FGFR3*, bone growth is slowed (22). Radiographs show narrow sacriosciatic notch, decreasing interpedicular distance cranial to caudally, flat acetabular roofs, short robust long bones, and metaphyseal flaring (Figure 5). Pseudoachondroplasia arises from a pathogenic variant in Cartilage Oligomeric Matrix Protein (*COMP*) that ultimately result in epiphyseal dysplasia (23). *COMP* pathogenic variants prevent export to the extracellular matrix and thus it is retained in chondrocytes leading to cell death and short stature. The extracellular matrix is also abnormal because of lack of COMP. Radiographs reveal abnormal epiphyses and vertebral endplates (Figure 5). LWD and Turner syndrome both result in short stature and mesomelic shortening due to haploinsufficiency or pathogenic variants in Short Stature Homeobox (*SHOX*) or its promoter region (24). Madelung deformity can be seen on radiographs. SHOX related skeletal dysplasias also frequently show downward displacement of the distal femur medial condyles on Xray (Figure 5). MED is a genetically heterogeneous group of epiphyseal dysplasias that tend to affect the extracellular matrix of the growing bone and present with late or abnormal epiphyses on Xray (Figure 5) (21). Short trunked dysplasias include those arising from type 2 collagenopathies (*COL2A1* variants). Type 2 collagen is important in epiphyseal growth and hyaline cartilage. Radiographs show delayed and abnormal epiphyses and flattened vertebrae (platyspondyly). It is also involved in other tissues of the body such as the vitreous (25, 26). In general, hands and feet are spared in collagen 2 dysplasias. *TRPV4* related dysplasias include spondylometaphyseal dysplasia Kozlowski type and metatropic dysplasia (20). *TRPV4* is also associated with Charcot Marie Tooth neuropathy and thus patients may present with nerve symptoms (27). Table 3 summarizes the genetics of the more common skeletal dysplasia diagnoses.

**Table 3:** Most common skeletal dysplasia diagnoses.

Clinical diagnosis	Associated gene	Inheritance	Distinguishing features that may be present
Achondroplasia	*FGFR3* 98% with p.Gly380Arg c.1138 G>A c.1138 G>C	AD 75%–80% are *de novo*	Micromelia, macrocephaly, midface hypoplasia
Hypochondroplasia	*FGFR3* Most common p.Asn540Lys c.162° C>A and c.162° C>G	AD 30% with unknown genotype	Mild micromelia, temporal lobe seizures, learning differences
Pseudoachondroplasia	*COMP*	AD	Micromelia, normocephaly, joint pain, significant joint laxity
Multiple epiphyseal dysplasia (MED)	*COMP, MATN* *COL9A1, COL9A2, COL9A3* *DTDST (SLC26A2)* *CANT1*	AD AR 15%–20% with unknown genotype	Joint pain, especially hips
Spondyloepiphyseal dysplasia congenita (SEDC) and other type 2 collagenopathies	*COL2A1*	AD	Short trunk, waddling gait Cleft palate club feet
Leri-Weill dyschondrosteosis (LWD)	*SHOX*	AD	Mild micromelia Mesomelia Madelung deformity
Diastophic dysplasia	*DTDST (SLC26A2)*	AR	Joint contractures, hitchhiker thumbs, club feet
Spondylometaphyseal dysplasia Kozlowski (SMD-K)	*TRPV4*	AD	Scoliosis Short trunk Pectus carinatum
Hypophosphatasia	*ALPL*	AR	Life limiting with small chest and respiratory failure to normal stature with increased fractures
X-linked hypophosphatemic rickets	*PHEX*	XLD	Leg bowing Metaphyseal flaring
Osteogenesis imperfecta	*COL1A1* or *COL1A2* for types I-IV	AD	Increased fractures, Blue sclerae

AD, autosomal dominant; AR, autosomal recessive; XLD, X-linked dominant.

Metabolic bone diseases that may present with short stature include hypophosphatasia, X-linked hypophosphatemic rickets, osteogenesis imperfecta, and hypoparathyroidism (28). Hypophosphatasia results from loss of function pathogenic variants in the *ALPL* gene which encodes tissue nonspecific alkaline phosphatase enzyme. Pathogenic variants cause inorganic phosphate to increase which ultimately inhibits bone and teeth mineralization (29). X-linked hypophosphatemia (XLH) is the most common heritable form of rickets and results from renal phosphate wasting due to loss of function of *PHEX* causing excess FGF23, resulting in impaired phosphate reabsorption and ending in hypophosphatemia and decreased conversion of vitamin D (10). The large majority of osteogenesis imperfecta (OI) arises from pathogenic variants in *COL1A1* or *COL1A2* which encode type 1 collagen. Type 1 collagen is the primary extracellular matrix protein in bone. The majority of the less common forms of OI result from abnormal type 1 collagen post-translational processing or collagen fibril assembly (30). Parathyroid hormone is a key regulator of the zone of endochondral hypertrophy and congenital hypoparathyroidism results in a metabolic bone disease characterized by hypocalcemia and radiological changes described as “stippled epiphyseal dysgenesis”. Standard treatment has focused on calcium and vitamin D administration (5, 12).

Osteoblasts which can be derived from a number of different cells, including growth plate chondrocytes, bone marrow stromal cells and quiescent bone-lining cells (31), are crucial in laying down the bone matrix which is composed primarily of type I collagen, and other extracellular proteins. The matrix is initially an unmineralized osteoid which then mineralizes through the accumulation of hydroxyapatite, an inorganic mineral with a lattice network of calcium and phosphate. Bone is constantly being remodeled with an intricate balance between osteoclastic and osteoblastic activity. There are a number of gene/endocrine regulatory mechanisms which maintain normal balance and any disruption can lead to reduced mineralization (osteopenia) or dysplasia and malformation. This homeostatic balance has been targeted in the osteopenic bone dysplasias including osteogenesis imperfecta, hypophosphatasia and physiological osteoporosis.

## Proposed evaluation

When faced with a child or adolescent with disproportionate short stature and you are considering an underlying skeletal dysplasia, it is best to start with a skeletal survey and key metabolic bone labs. A skeletal survey should include lateral and posterior-anterior (PA) spine, anterior-posterior (AP) pelvis, the extremities, and a lateral skull. It is frequently helpful to get the entire upper and middle segments of the extremity on one film to be able to assess for subtle rhizomelia or mesomelia. Ribs can be assessed on the PA spinal film. For labs we propose a general screen consisting of serum calcium, phosphorous, alkaline phosphatase, 25-OH vitamin D, magnesium, PTH, urine calcium, and urine phosphate.

## New and evolving treatments

**Figure 6:**
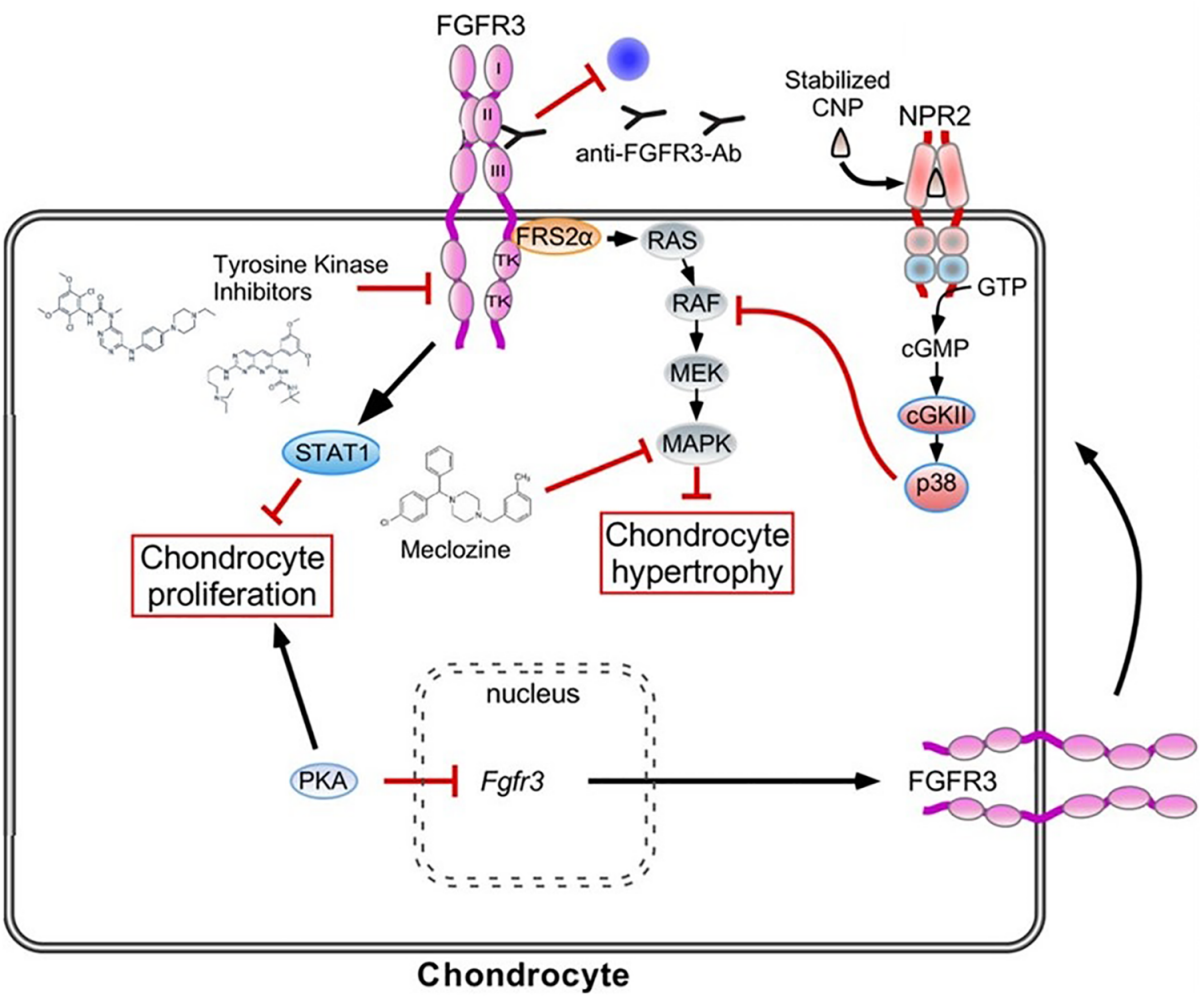
Achondroplasia is the most common genetic disorder of chondrogenesis. This figure outlines the primary pathway for its pathoetiology (gain of function of FGFR3 receptor which inhibits chondrocyte hypertrophy and maturation through the RAS pathway and inhibits chondrocyte proliferation through the STAT1 pathway) Therapeutic options include Vosoritide which is a synthetic CNP that in turn inhibits the RAS pathway. Additional CNP mediators are under investigation. The off-label use of Meclizine, a common anti-emetic is in clinical trial as a therapeutic option as it has been found to inhibit the RAS pathway. Antibodies targeted at the FGFR3 receptor are also under investigation in an attempt to reduce the overactive ligand that results in the Achondroplasia phenotype*.* (Figure adapted from Achondroplasia: Development, pathogenesis and Therapy) (32).

Achondroplasia arises from a pathogenic variant in *FGFR3* resulting in a gain of function in FGFR3 and downstream signaling causing decreased endochondral bone growth (Figure 6). Cyclic natriuretic peptide (CNP) negatively regulates the FGFR3 downstream RAS pathways, ultimately resulting in promotion of endochondral bone growth. Vosoritide, a subcutaneous injection of a CNP analogue, was approved by the FDA in November 2021 for increasing annualized growth velocity in children with achondroplasia 5 years and over with open growth plates (4). There have been some positive responses to vosoritide in patients with *FGFR3+*  hypochondroplasia as well as children with achondroplasia under 5 years of age.

Weekly CNP injections attached to an inert carrier (TransCon CNP) are also in clinical trials. Infigratinib is an oral inhibitor of the tyrosine kinase of FGFR3, thus inhibiting the downstream effects of FGFR3 activation. Both of these are in development and have successfully completed phase 2 clinical trials. TransCon CNP showed improvements in annualized growth velocity compared to placebo at 100 mcg/kg/week (8), and infigratinib showed significant gains in annualized growth velocity at 0.25 mg/kg daily (9). Anti-FGFR3 antibodies have also shown promise in pre-clinical studies.

Meclozine, an H1 inhibitor on the market for over 50 years, has also been found to downregulate FGFR3 signaling, possibly through phosphorylation of the kinase pathway (33). Mouse studies have been promising for increasing longitudinal growth and pharmacokinetic studies in children have shown safety (34, 35). Interestingly, meclozine also shows promise in a mouse model of X-linked hypophosphatemia (36). Further trials are ongoing.

There is preclinical evidence of improvement in growth in pseudoachondroplasia with anti-inflammatories such as resveratrol and aspirin that may alleviate endoplasmic reticulum (ER) stress (7). Spondyloepiphyseal dysplasia congenita (SEDC) arises from pathogenic variants in *COL2A1*. Preclinical research is in process regarding possible treatments.

Burosumab was approved by the FDA in June 2020 for treatment of X-linked hypophosphatemia (XLH) in patients over 1 year of age. Given that excess circulating FGF23 is the primary mediator of the osteomalacia, Burosumab was developed as a human IgG1 monoclonal antibody targeting FGF23. Patients receiving Burosumab had improved renal tubular phosphate reabsorption, decreased Thacher rickets scores (healing), improved height Z-scores, and improved 6 min walk tests. It is administered as a subcutaneous injection (SQ) every 2 weeks (11, 37).

**Figure 7:**
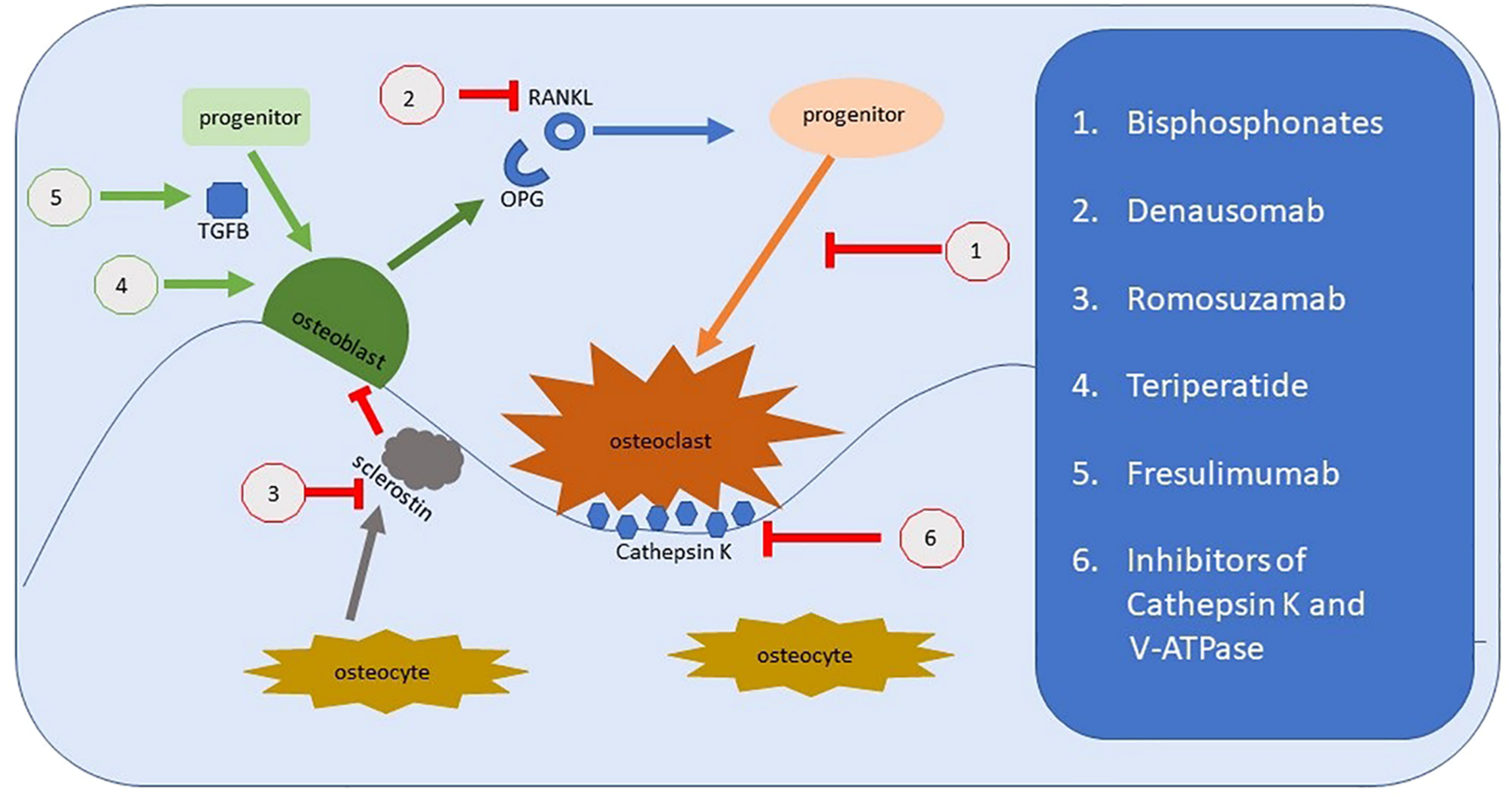
Representation of bone remodeling and the targets for therapeutic intervention. Bisphosphonates were the archetypal treatments to improve bone loss by reducing osteoclastic activity. There are several new medications that target alternate physiological pathways, but the end-point remains the same, to reduce the effect of osteoclasts. This includes newer medications under investigation which target Cathepsin K and the hydrogen pump, V-ATPase. Denausomab, Fresulimumab and Teriparatide all act by improving the osteoblast lineage.

Osteogenesis imperfecta (OI) is most frequently caused by pathogenic variants in *COL1A1* or *COL1A2,* ultimately decreasing the amount or quality of type 1 collagen. The general approach to managing OI is to treat the increased osteoclastogenic activity resulting from the incorporation of abnormal collagen 1 into the matrix. The approach takes one of 2 forms: inhibiting osteoclastogenesis or improving osteoblast “health” (Figure 7). The verdict is still out regarding bisphosphonates (38). They appear to improve fracture rates and function in children when initially given, but not in adults. Bone density is improved, but fracture rates and quality of life remain stable. Bisphosphonates inhibit osteoclast function. A current trial using Teriparatide, a human PTH analogue, which would increase osteoblast activity is underway in the UK. Other treatments include Denosumab a RANKL inhibitor. RANKL is involved in maturation of osteoclasts and thus ultimately decreases osteoclast function. Other treatments in development include TGFβ antibodies (12).

Hypophosphatasia (HPP) treatment has changed with the approval of asfotase alfa (29). It was approved by the FDA in 2015 and replaces alkaline phosphatase, thus decreasing inorganic phosphate accumulation and improving bone and teeth mineralization. It has been life changing for severe disease. It is administered in a daily SQ injection. Gene therapy studies are underway (39).

Parathyroid hormone regulates calcium homeostasis. Standard treatment for congenital hypoparathyroid has focused on administration of calcium and vitamin D. Unfortunately, conventional treatment has its own complications and doesn't restore PTH effects in the kidneys. Recombinant human parathyroid hormone is approved by the FDA for adults (40), but use in pediatric patients is still rare given a theoretical risk of osteosarcoma. Case reports have shown it is safe in children without osteosarcoma described (41).

## Conclusion

Short stature skeletal dysplasias are frequently seen in the pediatric endocrinology clinic. They commonly present with disproportion or other noteworthy medical and family history. If you are concerned about a possible skeletal dysplasia, the initial evaluation should include a skeletal survey and metabolic bone labs. If these are concerning, consultation with a genetic specialist is warranted. There are new treatments available for skeletal dysplasias, especially achondroplasia, hypophosphatasia and some additional metabolic bone diseases. Pediatric endocrinologists will increasingly find themselves seeing these patients in order to monitor growth and manage therapeutic interventions. Pediatric endocrinologists and geneticists must collaborate for best outcomes and optimal patient care.
